# Space Regaining Made Easy: The Case of a Severely Infraoccluded Primary Molar

**DOI:** 10.1155/2019/6916839

**Published:** 2019-06-11

**Authors:** Daniele Garcovich, Riccardo Aiuto, Milagros Adobes Martin

**Affiliations:** ^1^Universidad Europea de Valencia and the Department of Pediatric Dentistry, CEU-UCH, Spain; ^2^University of Milan-Department of Oral Rehabilitation, Istituto Stomatologico Italiano, Italy; ^3^Universidad Europea de Valencia and the Department of Pediatric Dentistry, Universidad de Valencia, Spain

## Abstract

Infraocclusion of deciduous molars is a quite common but challenging clinical situation that a paediatric dentist has to face in his everyday practice. This anomaly can lead to space loss, eruption disturbances of the permanent successor, and deformation of the occlusal plane. A case of a severely infraoccluded primary molar is presented. The treatment was carried out using a compressed NiTi wire applied only to the adjacent teeth. In three months, the space was recovered, and the infraoccluded temporary molar was extracted. After one year, the permanent successor erupted without any complication. The technique presented can be considered minimally invasive, and it involves cost- and time-efficient mechanics.

## 1. Introduction

The infraocclusion is reported with variable and maybe misleading nomenclatures, such as submerged tooth, ankylosed tooth, secondary retention, incomplete eruption, impaction, depression, intrusion, and a shortened tooth [1]. This anomaly, affecting mostly the primary molars in different degrees, is a clinical situation that paediatric dentists frequently have to deal with. The infraocclusion has a variable prevalence among different populations ranging from 1.3% to 38.5% [2]. A prevalence of 21.8% has been reported recently in a sample of Spanish children [3].

It is important to stress that although infraocclusion is often associated with ankylosis, the ankylosis is not always the cause of infraocclusion. The molar can be infraoccluded but not ankylosed. Many theories have been proposed to explain its etiopathogenesis, and genetic, epigenetic, and environmental factors are called into play. Probably, all these factors have a role in determining the aetiology, the clinical expression, and the degree of severity of the infraocclusion. Moreover, the infraocclusion can coexist and be related to other dental anomalies such as hypodontia, ectopic canines, and peg-shaped lateral incisor [4].

## 2. Case Report

An eight-year-old boy was referred to our department (Clinica Odontologica de la Universidad Europea de Valencia) for an orthodontic consultation. The patient had a Class I canine occlusion on both sides, increased overjet, and moderate crowding in the anterior segments in both the upper jaw and the lower jaw. The lower-left first molar (4.6) appeared tipped forward in the space of the second deciduous molar (8.5).

**Figure 1:**
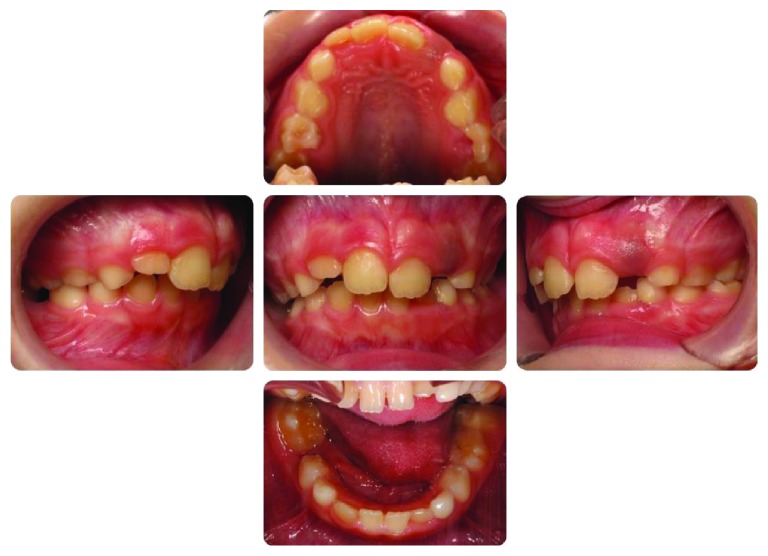
Intraoral photographs of the case at first examination. The second deciduous molar (8.5) is not visible at a clinical examination.

Severe caries were affecting the second deciduous molars in the upper jaw (5.5 and 6.5), and ectopic eruption of the first permanent molars (1.6 and 2.6) could be diagnosed from a clinical examination (Figure 1).

**Figure 2:**
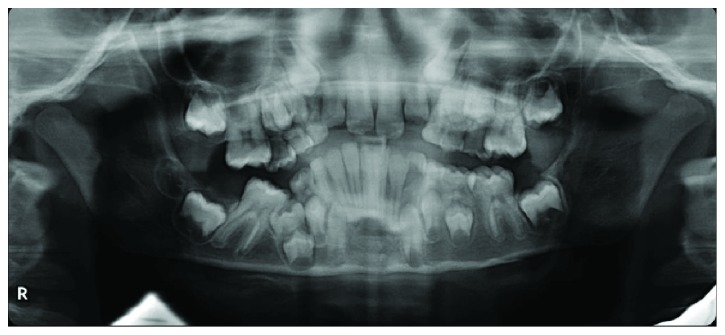
Orthopantomogram of the case at first examination. The second deciduous molar appeared severely infraoccluded. The first molar (4.6) was severely inclined mesially.

A radiographic examination was carried out to check the eruption process and the maturation stage. From the orthopantomogram (OPG), the second deciduous molar on the right side (8.5) appeared to be totally submerged (Figure 2).

On the basis of the clinical and radiologic assessments, the extraction of the 8.5 was planned in order to prevent impaction of the permanent successor. An appropriate space regaining strategy had to be carried out to gain access to the infraoccluded tooth and facilitate its extraction. Due to the high risk of caries of the patient and not wanting to rely on his collaboration, we designed minimally invasive mechanics to carry on the space regaining procedure.

A band with a double buccal tube was fitted on the permanent molar, and a .014-inch round NiTi wire (Highland Metals Inc., Franklin, IN) was compressed in between the first permanent molar and the first deciduous molar using the described procedure.

**Figure 3:**
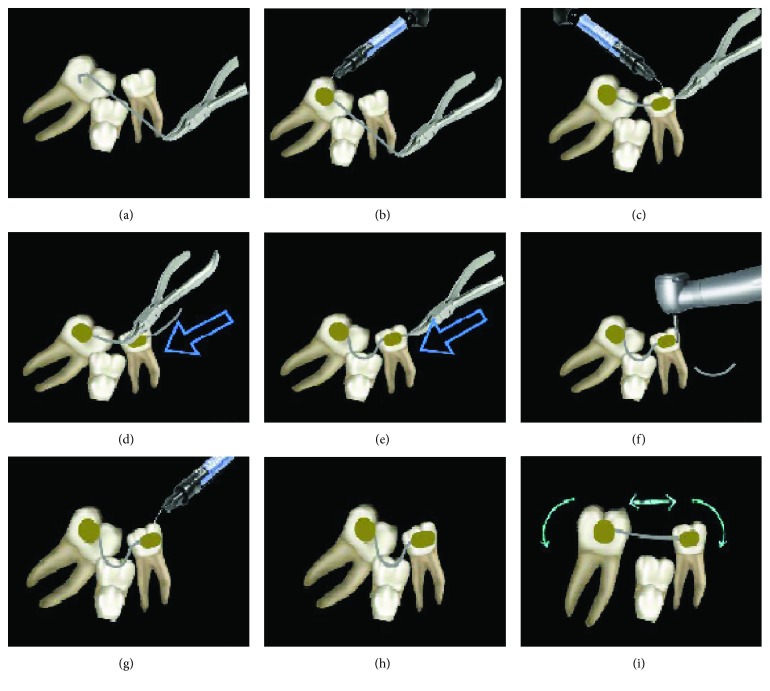
Steps of the clinical application of the compressed NiTi wire.

The following are the steps of the clinical procedure (Figure 3):

1. A piece of .014 NiTi wire is cut and bonded with flowable composite to the buccal surface of the first permanent molar. It is very important to bond the wire perpendicular to the main tooth axis. (In this case, a band was used to improve stability, and the buccal tube was perpendicular to the main tooth axis) (Figures 3(a) and 3(b))
2. The arch wire is then bonded to the surface of the mesial tooth, and also in this case, it is very important to bond the wire perpendicular to the main tooth axis to improve the uprighting moment of the mechanics. To avoid the bond between the flowable composite and the archwire, the latter is isolated with liquid Vaseline (Figure 3(c))
3. The wire is then compressed by pulling it down and distally, and then, a loop is formed between the mesial tooth and distal tooth (Figures 3(d) and 3(e))
4. The wire extending from the mesial tooth is then cut flush and sealed with some flowable composite to prevent it from coming out from the mesial end (Figures 3(f) and 3(g))
5. The compressed wire gently recovers the original straight shape delivering a reciprocal sagittal force and two uprighting moments on the mesial and distal units of the system (Figures 3(f) and 3(g))

**Figure 4:**
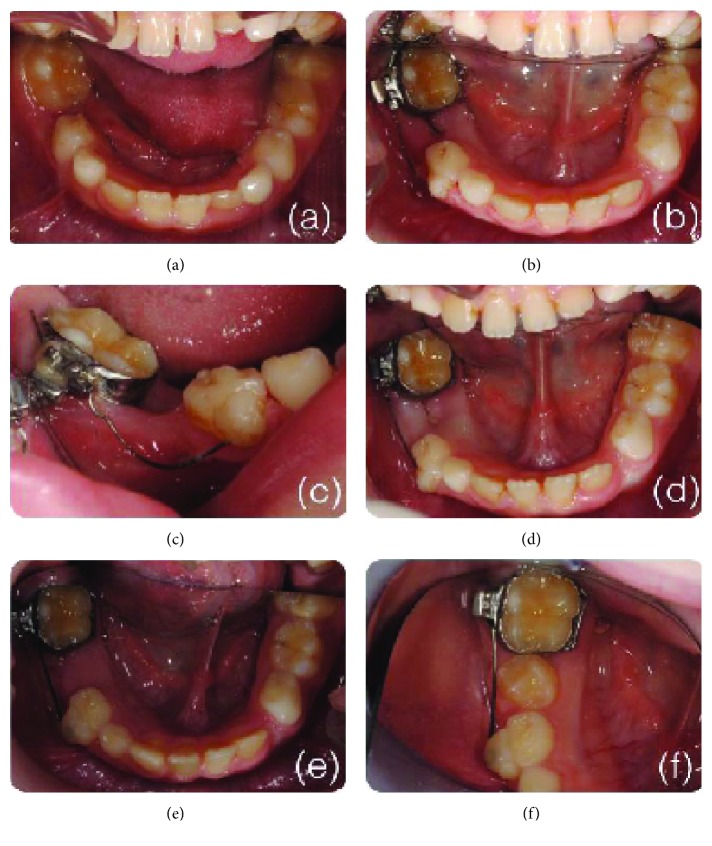
Clinical stages of the space regaining process: (a) first clinical examination; (b, c) after one month with the mechanic in place; (d) after three months before the extraction of the deciduous molar; (e, f) retention phase with a stiff wire until the eruption of the second premolar.

The mechanics did not need reactivation, and after three months, the space was recovered and the extraction of the second deciduous molar was performed (Figure 4(d)).

At this stage, a stiffer .018 Green Australian archwire (A.J. Wilcock Scientific and Engineering Company, Whittlesea, Victoria, Australia) was bonded instead of the NiTi one in order to maintain the space for the eruption of the second premolar (Figures 4(e) and 4(f)).

**Figure 5:**
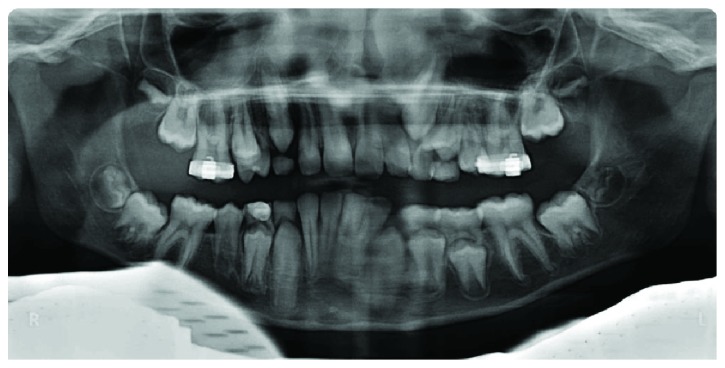
Orthopantomogram of the case at the end of the treatment. The second premolar (4.5) has successfully erupted, and the first molar (4.6) presents a correct inclination.

One year later, the second premolar erupted. The final OPG shows the eruption of the second premolar, the uprighting of the first permanent molar (with no side effects on the eruption of the first premolars), and the canine on the same side (Figure 5).

## 3. Discussion

Many appliances have been proposed to regain space and favour the normal eruption process in patients with eruption disturbances; some of them were removable and some fixed [5]. The Halterman appliance is one of the classical solutions to recover space by means of a distal tipping and has been used to correct ectopic eruption of the permanent first molar. This fixed appliance relies mainly on the primary second molar for retention and can have a detrimental effect on this tooth, which is, in most of the cases, already affected by a certain degree of root resorption. Moreover, the bulky distally extended loop can be bitten and displaced and cause tissue impingement [6]. A simple alternative for space regaining could be a system based on fixed appliances, such as sectional wires with an open coil spring, as suggested by the American Academy of Pediatric Dentistry (AAPD) reference manual [7]; however, this option is not side effect free. The open coil should be compressed between the two brackets that can debond due to the occlusal contacts, diet, or habits. The coil itself offers retention to dental plaque and food debris, hindering the normal hygiene. Last but not least, the system needs some extra wire to extend mesially or distally, acting as a track for the two units to slide on. This extra wire can be dislodged, halting the mechanic or impinging in the tissues. Mitsuhata et al. in 2014 reported a system for space regaining in the lower jaw made by a lingual arch extending from the second deciduous molars towards the distal with the target of tractioning the permanent molar distally from a button bonded to its crown [8]. It should be highlighted that if the permanent second molar is not yet erupted and its crown is located at the midroot level of the permanent first molar, the anchorage requirements are low. If anchorage is not a main concern, all the systems involving complicated and bulky structures are not needed. In the case reported, the first deciduous molar, despite the incipient root resorption, is deemed to be stable enough to stand the force exerted by the compressed wire without side effects. The displacement of this tooth would not be a problem, since the tooth constituted a so-called “free anchorage” unit. “Free anchorage indicates that no ‘price' has to be paid in terms of undesirable forces on teeth belonging to the anchorage unit, if the reactive forces are transferred to teeth which are to be extracted according to the treatment plan” [9].

For all the aforementioned side effects, a minimally invasive approach is necessary. Removable appliances can be less invasive and allow the maintenance of a better hygiene standard, but they strongly rely on patient compliance. According to Shah in 2017, the compliance with removable orthodontic appliances is suboptimal [10]. Patients, generally, wear appliances for considerably less time than stipulated and self-reported. The lack of compliance can derive in a suboptimal treatment result or treatment failure. The age is also strongly related to compliance, and the younger the patient, the lesser the adherence is to the treatment prescriptions and the appliance wear time [11]. Moreover, fixed and removable appliances can cause a change in the oral microbiota and biofilm that can be related to an increased risk of caries [12]. The fixed appliances proposed for the treatment of eruption anomalies can be bulky and somewhat complex. The technique reported by the authors is a minimally invasive technique that does not rely on patient compliance and proven to be cost and time efficient since it can be manufactured chairside with the materials that are currently available in an actual dental office.

## 4. Conclusions

Many clinical situations requiring a different amount of space recovery are quite common in paediatric dentistry. An ideal appliance should be minimally invasive, little plaque retentive, effective, easy to manage, and compliance-free. A simple device was designed to address all the previous requirements. The appliance proved to be effective and side effect-free.
